# Subacute Posterior Inferior Cerebellar Artery Stroke Radiographically Mimicking Lhermitte-Duclos Disease

**DOI:** 10.7759/cureus.31381

**Published:** 2022-11-11

**Authors:** Yusuf Mehkri, Jordan Poe, Imran Nasrullah, Jairo Hernandez, Natalie Buchwald, Justin De Prey, Mehmet Albayram, Hans Shuhaiber

**Affiliations:** 1 Medicine, University of Florida College of Medicine, Gainesville, USA; 2 Surgery, University of Florida College of Medicine, Gainesville, USA; 3 Neurology, University of Florida College of Medicine, Gainesville, USA; 4 Radiology, University of Florida College of Medicine, Gainesville, USA

**Keywords:** neuro-imaging, neurology case report, cerebellar infarct, cerebrovascular stroke, lhermitte-duclos disease

## Abstract

Lhermitte-Duclos disease (LDD) is a rare cerebellar lesion characterized by a hamartomatous lesion of the cerebellum. Mainly diagnosed by MRI, the clinical presentation is usually made of neurological symptoms. Modern neuroimaging techniques such as MRI have led to accurate diagnosis of this disease in both its pre- and post-operative periods. We present the case of a 68-year-old male with a past medical history of cardiac stenting and coronary artery disease who originally presented to the emergency department as a transfer for evaluation of possible obstructing hydrocephalus and left posterior inferior cerebellar artery (PICA) infarct. Based on the clinical presentation and imaging, the favored diagnosis of his left cerebellar abnormality was LDD rather than an unusual acute/subacute infarct or a metastatic lesion. The rapid progression of symptoms with rapidly progressive cytotoxic edema on serial CTs helped exclude LDD, which is nearly always more of a chronic process. The classic neuroimaging findings and clinical presentation of LDD are also discussed.

## Introduction

Lhermitte-Duclos disease (LDD) is a rare neurological disease characterized by a proliferative benign tumor, called a dysplastic gangliocytoma, which leads to a hamartomatous lesion of the cerebellum [1,2]. Resulting from the thickening and hypermyelination of the outer molecular layer of the brain, LDD produces an abnormal development and unilateral hemispheric expansion of the cerebellum and associated parts of the brain [3]. LDD itself is believed to be a pathognomonic symptom of a hereditary cancer called Cowden’s disease, which is a multi-system autosomal dominant disorder characterized by both benign and malignant neoplasms [4]. Historically, documentation of the disease prevalence had been limited until its discovery by Lhermitte and Duclos in 1920, but around 230 cases have since been reported throughout historical medical literature [2]. Furthermore, LDD manifests most commonly in the third and fourth decades of life; however, it has been observed in young adults, indiscriminate of sex or race [2,3]. Modern neuroimaging techniques such as magnetic resonance imaging (MRI) have led to a predictable diagnosis of this disease in both its pre- and post-operative evaluation, likely resulting from its general classification of low malignancy grade as well as a slowly progressive malformative-dysplastic disorder [1]. 

## Case presentation

**Figure 1:**
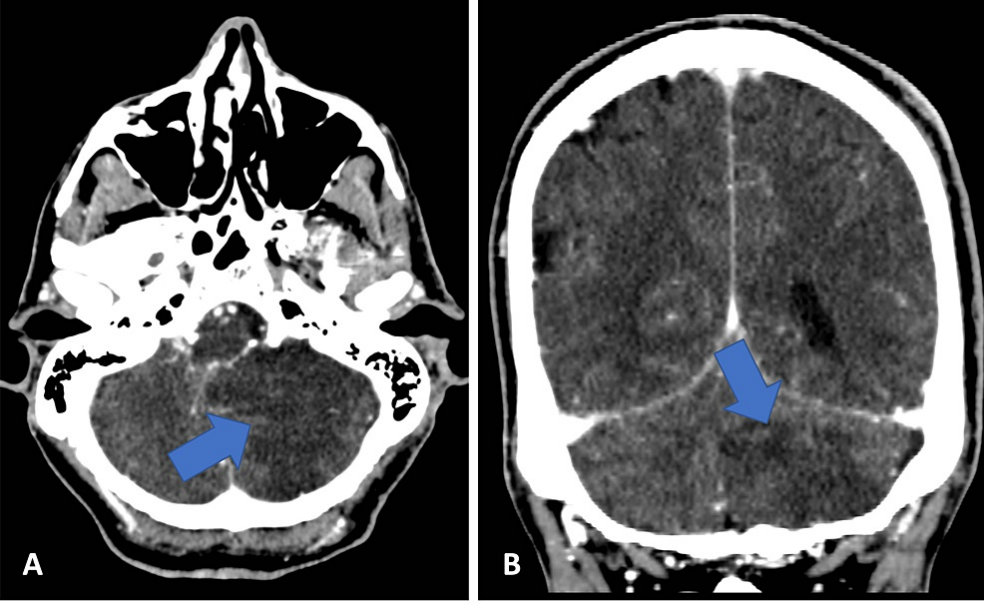
Axial (A) and coronal (B) images reveal triangular hypodensity in the left cerebellum.

This case is of a 68-year-old male with a past medical history of atrial fibrillation, coronary arteriosclerosis, and cardiac stenting who presented to the emergency department (ED) as a transfer for evaluation of a cerebellar mass vs. stroke lesion. Six days prior to admission, he presented to an external care facility with complaints of dizziness, chest pain, and syncope, and underwent cardiac stenting following abnormal electrocardiogram findings. Negative troponins later revealed a diagnosis of pericarditis rather than ST-elevation myocardial infarction (STEMI), and stents were placed for intraoperative findings of stenosis. He declined anticoagulants for atrial fibrillation and was discharged on oral aspirin and clopidogrel, but returned the following day with new intractable headache, nausea, and vomiting. A non-contrast head computed tomography (CT) taken two days prior to admission revealed a moderate-sized area of decreased attenuation in the left cerebellar hemisphere (Figure 1).

Differential diagnoses included infarction of indeterminate age versus underlying mass and the patient was transferred to our facility for further workup. He arrived somnolent but was easily arousable and conversant, able to give a history, and endorsed moderate headache, diplopia, and nausea. Vital signs were within normal limits and the patient appeared well, awake, and oriented to person, place, time, and events. Neurologic examination at time of admission revealed left sixth cranial nerve (CNVI) palsy and left horizontal beating nystagmus. Pupils were equal, round, and reactive to light, and no other cranial nerve deficits were found. Speech was fluent without obvious language deficits. Motor and sensory exams were normal. All reflexes were 2+ bilaterally and negative for Babinski, Clonus, and Hoffman. Cerebellar testing revealed left dysmetria and absent pronator drift.

**Figure 2:**
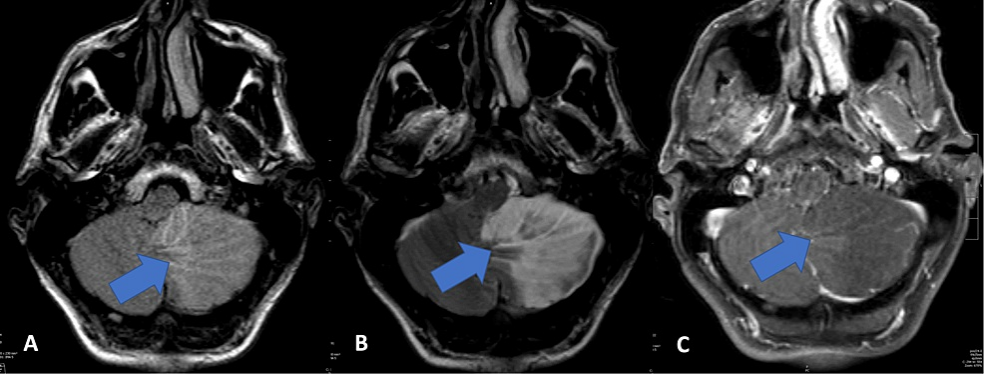
Axial fluid-attenuated inversion recovery (FLAIR) (A), T2 (B), and axial T1 (C) post contrast series reveal widened cerebellar folia with a striated/laminated appearance in the left cerebellum without contrast enhancement.

An MRI with and without contrast showed an intra-axial, expansile abnormality suspicious for dysplastic gangliocytoma in the left cerebellar hemisphere (Figure 2). The margins were ill-defined and measured approximately 5.3 x 5.1 cm with diffuse restricted diffusion signal. Rightward mass effect can be seen on the midbrain, pons, and medulla with near complete effacement of the fourth ventricle and lower perimesencephalic cisterns. Tonsillar herniation and obstructive hydrocephalus were developing.

Hypointense signal abnormalities indicated the presence of hemosiderin likely consistent with petechial hemorrhage. Additionally, two foci of abnormal enhancement could be seen in the left posterior parietal lobe at the grey-white junction, with the largest measuring 1.1 cm. This supratentorial finding was unusual and may have represented subacute cerebrovascular accidents (CVAs). Radiographically, at this time, a dysplastic gangliocytoma as seen with LDD was the favored differential diagnosis for the patient’s left cerebellar abnormality rather than an acute or subacute CVA involving the anterior or posterior inferior cerebellar arteries. His clinical presentation, however, appeared more consistent with Wallenberg Syndrome caused by a posterior inferior cerebellar artery (PICA) infarct.

**Figure 3:**
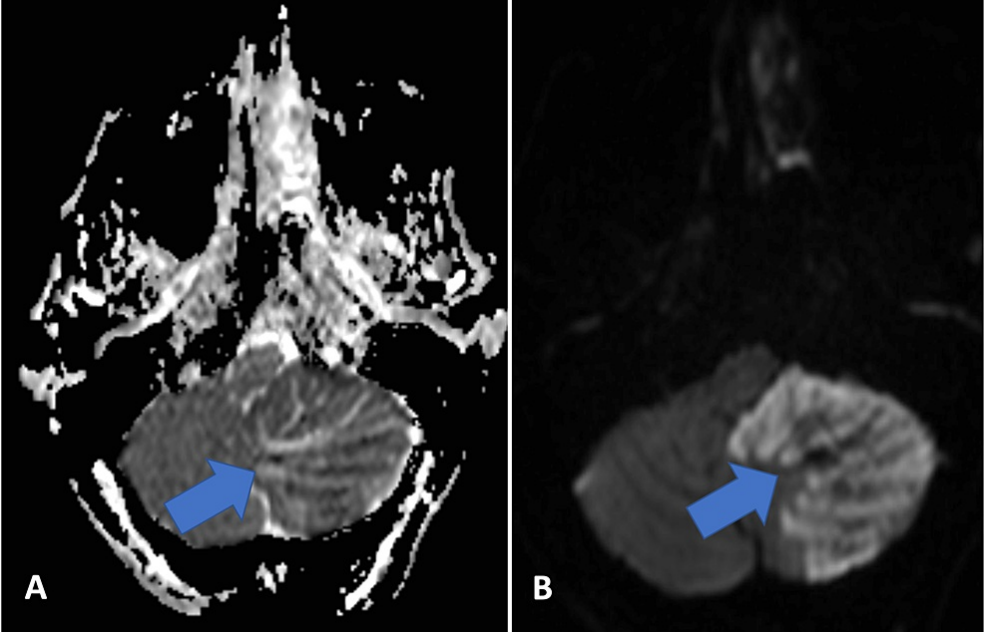
Axial apparent diffusion coefficient (ADC) map (A) and diffusion-weighted imaging (DWI) image (B) reveal striated/laminated appearance in the left cerebellum without significant diffuse diffusion restriction. The cerebellar cortex show hyperintensity most likely due to T2 shine-through effect.

Repeat non-contrast head CT demonstrated progressive cytotoxic cerebral edema in the inferior aspect of the left cerebellar hemisphere including the cerebellar vermis and left side of the tonsil. Anterior displacement of the brainstem and worsening tonsillar herniation were observed without evidence of hemorrhagic transformation. Follow-up CTA of the head and neck revealed patent PICAs bilaterally with no evidence of intracranial large vessel occlusion, flow-limiting stenosis, or aneurysm in the anterior or posterior circulation. Small calcifications causing approximately 50% stenosis were seen at the extracranial origin of the left vertebral artery. CT perfusion study found no abnormalities in any large vascular territories supplied anterior circulation. An increased time to peak and associated decreased cerebral blood volume was revealed in the left inferior cerebellum consistent with a subacute infarction in a PICA territory (Figure 3). 

The patient’s condition continued to deteriorate and he became increasingly difficult to arouse (Glasgow Coma Scale (GCS) 13 and NIH Stroke Scale (NIHSS) 4). At this point, his presentation and imaging findings were most consistent with a subacute infarct in the left PICA territory. Due to declining mental status and progression of cytotoxic edema, the patient underwent a suboccipital craniectomy and C1 laminectomy for decompression of the brainstem with placement of an external ventricular drain (EVD). After a prolonged EVD clamping trial during his hospitalization, CT scans of his brain showed no ventriculomegaly or increased size of his post-operative pseudomeningocele. He was discharged with no evidence of cerebellar ataxia or dysmetria.

At his one-month follow-up, he reported symptoms concerning for hydrocephalus, including lethargy, confusion, and nausea. On exam, he was found to have mild dysmetria on the left upper extremity. A head CT showed significant ventriculomegaly and expansion of his known pseudomeningocele compared to scans from his hospitalization. There was no evidence of cerebral spinal fluid leak. He was directly admitted from the clinic and underwent placement of a ventriculoperitoneal shunt approximately two weeks later for management of obstructive hydrocephalus.

## Discussion

Here, we have presented a case of a subacute PICA stroke which radiographically mimicked LDD; however, the rapid progression of symptoms with rapidly progressive cytotoxic edema on serial CTs helped to differentiate it from LDD, which is nearly always more of a chronic process. While some patients are asymptomatic, LDD most commonly presents with chronic and progressive symptoms, with symptoms typically developing after a few months to more than 10 years prior to diagnosis [1,5]. LDD most commonly presents with cerebellar ataxia, dysarthria, cranial nerve dysfunction, and psychiatric symptoms [6]. In more severe cases, obstructive hydrocephalus and intracranial hypertension may develop, leading to macrocephaly and bilateral papilledema [2,3,7]. These symptoms may be explained by structural changes in the cerebellum region of the brain as well as morphological changes in the biological makeup of affected neurons [5]. Structural changes have been associated with the loss of neurotransmitter function within the cerebellum. Key to this has been the absence of benzodiazepine receptors in the hamartomatous lesion of the cerebellum, which also points to loss of function of relevant neurotransmitters and their receptors in surrounding brain tissue [2]. Furthermore, neurons found in LDD lesions have been characterized as having a larger and rounder morphology compared to normal brain tissue [5].

Imaging is commonly a diagnostic tool when assessing the disease onset of LDD, as MRI is primarily used to detect the dysplastic gangliocytoma [8]. Cerebral MRIs have been used to capture images of the cerebellar lesions, which can rarely enhance heterogeneously and are surrounded by a noticeable edematous reaction [5]. Particularly in T2-weighted images, the lesions appear with high-signal intensity as well as a striated pattern with isointense bands in areas showing high intensity, which have been attributed to the morphological changes in the granular and intermolecular cell layers characteristic of LDD associated with the atrophy of cerebellar white matter [1,5]. On the other hand, T1-weighted images show little to no enhancements, suggesting light disturbances in the blood-brain barrier [5]. In the case of LDD, MRI is primarily preferred over CT, which cannot capture potentially hypoattenuated images of LDD’s characteristic hamartomatous lesion [1]. In other cases, the diagnosis of LDD can be accounted for in expected radiological findings that can sometimes lead to bypassing the need for a biopsy in most cases [3]. Cerebellar stroke may radiographically mimic LDD, especially at first glance, but it's always important to consider these imaging findings in the appropriate clinical context. When symptoms are severe or debilitating, surgery is the primary treatment method. Specifically, ventricular shunting may be used to manage obstructive hydrocephalus, and a sub-occipital approach is used for tumor resection [3,5]. Otherwise, symptomatic (vertigo, headache, etc.) treatment of LDD is another commonly preferred approach, as the absence of observed tumor margins during relevant surgical procedures constitutes a major technical problem [1,5].

## Conclusions

Our case displays a cerebellar stroke that radiographically mimicked LDD, especially at first glance, but the clinical context helped narrow the differential diagnosis. Imaging is used to diagnose LDD, and the rapid progression of disease on serial CTs, as well as patient history, revealed the underlying stroke in this patient. This case highlights the importance of disease progression in the context of possible LDD.
